# Factors associated with catastrophic total costs due to tuberculosis under a designated hospital service model: a cross-sectional study in China

**DOI:** 10.1186/s12889-020-09136-z

**Published:** 2020-06-26

**Authors:** Tianchi Yang, Tong Chen, Yang Che, Qin Chen, Dingyi Bo

**Affiliations:** 1 Institute of Tuberculosis Prevention and Control, Ningbo Municipal Center for Disease Control and Prevention, Ningbo, Zhejiang China; 2 Division of Medical Insurance, Ningbo Huamei Hospital, University of Chinese Academy of Sciences, Ningbo, China; 3 Institute of Infectious Disease Control, Haishu District Center for Disease Control and Prevention, Ningbo, Zhejiang China

**Keywords:** Tuberculosis, Catastrophic total costs, Designated hospital, Factors

## Abstract

**Background:**

Certain districts and counties in China designated local general hospital as the designated hospital for tuberculosis (TB) management after the promulgation of the Law of Practicing Physicians in 2009. To our knowledge, there is limited research on catastrophic payments of TB patients under this service model, often with inconsistent conclusions. In addition, there has been no published studies from China using the updated 2018 World Health Organization (WHO) definition of catastrophic total costs due to TB. This study used the latest criterion recommended by the WHO to analyze the incidence of catastrophic total costs for households affected by TB under the designated hospital model and explore its influencing factors.

**Methods:**

A cross-sectional analysis was carried out in all ten designated hospitals in Ningbo, China. Eligible pulmonary TB cases confirmed by sputum culture of *Mycobacterium tuberculosis* were recruited and surveyed from September 2018 to October 2018. We evaluated catastrophic total costs using total costs for TB treatment exceeding 20% of the household’s annual pre-TB income. A sensitivity analysis was performed while varying the thresholds. The least absolute shrinkage and selection operator (LASSO) regression were applied to select variables, and multiple logistic regression analysis were used to identify the determinants of catastrophic total costs.

**Results:**

A total of 672 patients were included, with a median age of 41 years old. The rate of catastrophic total costs of surveyed households was 37.1%, and that of households affected by MDR was 69.6%. Medical cost accounted for more than 60% of the total cost. 57.7% cases were hospitalized. The hospitalization rates of patients with no comorbidities, no severe adverse drug reactions, and rifampin-sensitive TB were 53.9, 54.9, and 55.3%, respectively. Patients in the poorest households had the highest hospitalization rates (Q1:54.8%, Q2:61.4%, Q3:52.2%, Q4:49.5%, Q5:69.7%, *P* = 0.011) and the highest incidence of severe adverse drug reactions (Q1:29.6%, Q2:19.6%, Q3:28.0%, Q4:33.7%, Q5:35.3%, *P* = 0.034). Factors such as elderly, minimum living security, unemployed before or after illness, poor economic status, seeking medical care outside the city, hospitalization, absence of local basic medical insurance coverage and MDR were positively associated with catastrophic costs.

**Conclusion:**

Substantial proportions of patients and households affected by pulmonary TB faced catastrophic economic risks in Ningbo, China. The existing policies that focus on expanding the coverage of basic medical insurance and economic protection measures (such as cash transfers to compensate low-income households for direct non-medical costs and income loss) might be insufficient. Tailored program that mitigate inappropriate healthcare and address equity of care delivery are worthy of attention.

## Background

Tuberculosis (TB) is one of the major worldwide infectious diseases, and it remains an urgent public health issue in developing countries [1]. Poverty increases the risk of TB, impacts of TB on wage and earning power further exacerbates impoverishment [2, 3]. Since 2005, the Chinese government has implemented a free TB-care policy that all pulmonary TB patients are provided with free sputum smear tests, chest X-ray examinations, and anti-TB drugs [4, 5]. Theoretically, patients do not need to pay out-of-pocket expenses for the diagnosis and treatment of TB.

The “Law of Practicing Physicians” promulgated by Chinese government in 2009 stipulates that public health doctors are not qualified to provide clinical care. In response to the regulations, each administrative district or county has designated a general hospital to set up a TB clinic to diagnose and treat TB within its jurisdiction. Other hospitals transfer suspected TB cases to designated hospitals, and the institutions for disease control and prevention no longer provide TB clinical services. TB patients treated at designated hospitals expressed concerns on the high level of out-of-pocket expenses after the service model changed [5–7]. Some patients interrupted or discontinued standardized treatment due to cost concerns [6].

To date, there have been few studies on catastrophic health expenditures (CHE) of TB patients under the designated hospital model in China, and the conclusions are not consistent [8, 9]. For example, Zhou C, etc. found that hospitalization is an important determinant of the occurrence of CHE [9], while in a study by Duan et al., despite observed strong association between hospitalization and CHE, the association was not statistically significant after adjusting for potential confounding factors (aOR:1.342, 95%CI: 0.950–1.897) [8]. The conflicting findings from two studies could probably resulted from the rates of patients participating in the New Cooperative Medical Scheme, an insurance specifically designed to cover more disadvantaged rural population. Because place of residence and type of health insurance scheme are highly confounded with inpatient service utilization patterns, the difference in insurance status distributions likely caused conflicting results [8, 9]. However, Li X, etc. pointed out that despite the limited benefit, patients participating in this scheme had a lower incidence of CHE [10]. In addition, since the World Health Organization (WHO) proposed catastrophic total costs, that is, total costs exceeding 20% of a household’s pre-TB annual income as the criteria for catastrophic payments in 2018, no relevant research reports from China have been seen.

Here, this study used the latest criterion recommended by the WHO to analyze the incidence of catastrophic total costs for households affected by TB under the designated hospital model, and explore its influencing factors.

## Methods

### Study sites

This study was conducted in ten counties of Ningbo Municipality, China. Ningbo is one of the most economically developed cities on the east coast of China, near Shanghai, with a population of 5.7 million permanent residents and 4.3 million floating population. There are ten designated hospitals for TB in the city, covering every district and county. The policy stipulated that from January 2016, all active TB patients and suspected cases were required to be referred to TB-designated hospitals for treatment. In 2017, the annual TB notification rate in Ningbo was 48.9 per 100,000, at a lower-middle level in the country.

According to the requirements of the National Tuberculosis Control Program (NTCP), pulmonary tuberculosis cases and suspected cases are diagnosed and treated in designated hospitals. Pulmonary tuberculosis treatment is carried out in accordance with the chemotherapy regimen recommended by the WHO or the personalized regimen made by clinicians. Generally, outpatient treatment is adopted, but complicated TB cases, patients with severe comorbidities or serious adverse drug reactions, addition to MDR cases required TB care under in-patient settings. MDR patients are referred to the municipal designated hospital for treatment, and are generally required to be hospitalized during the intensive period until the sputum culture turns negative. All patients are followed up regularly by community doctors throughout the treatment, including the intensive period once every 10 days and the continuation period once a month. During each visit, the community doctor will collect the patient’s medical expenses receipts, which are used to determine the amount of subsidies to the patients after treatment.

### Study design

A cross-sectional survey was carried out in all ten counties of Ningbo from September 1 to October 31, 2018. Due to the fact that extrapulmonary TB and tuberculous pleurisy were not covered by the NTCP during the study period, and there were overdiagnosis and misdiagnosis of clinically diagnosed cases, the study included only pulmonary TB cases confirmed by sputum culture. The pilot survey showed that the prevalence of catastrophic costs due to TB was about 40%. The study set a relative precision of 0.1, α error of 0.05, and an expected non-response rate of 10%. Therefore, the minimum sample size was 550. Until reaching our predetermined sample size, we consecutively recruited all sputum culture-positive pulmonary TB cases that met the following inclusion criteria from ten designated hospitals: (1) registered in designated hospitals in Ningbo between January 1, 2017, and December 31, 2017; (2) patients with rifampicin-susceptible TB who had finished treatment or patients with rifampicin-resistant TB who had been treated for at least 12 months.

### Data collection

Trained investigators interviewed each participant face-to-face using a modified version of the WHO patient cost tool [11]. The questionnaire included the demographic and socioeconomic status (age, gender, education, health insurance, household income and expenditure, etc.), health care seeking behavior (hospitalization, medical care outside the city, patient/diagnosis delay, etc.), clinical features (type of disease, adverse drug reactions and comorbidities, etc.), costs of TB diagnosis and treatment (medical cost, direct non-medical cost and income loss).

Direct medical costs refer to out-of-pocket (OOP) payments for medical services (consultation, medicine, and other medical procedures), net of any reimbursements. Direct non-medical costs refer to OOP payments related to accommodation, transportation, and food etc., net of any reimbursements. Indirect costs refer to income loss of a household caused by TB during the TB episode, estimated by self-reported household annual pre-TB income minus self-reported household annual income during TB treatment. The costs were ascertained from the onset of TB symptoms (the occurrence of cough, sputum production more than 2 weeks, or hemoptysis, blood-tinged sputum, fever or chest pain, etc.) to the end of treatment or investigation. Medical costs came from medical receipts collected by community doctors. The self-report was used to collect direct non-medical expenses and indirect costs, as well as a household income and living expenses. Costs and incomes were converted to United States Dollars (US$) using the average currency exchange rate during the study period (US$1 = 6.75 Chinese Yuan).

### Definitions

#### Household’s annual income

A household’s annual income refers to the total income of all members of a household for 1 year. The household’s annual pre-TB income means the household annual income in the year before diagnosis with TB.

#### Household’s annual capacity to pay

A household’s annual capacity to pay is the total annual income of a household minus the cost of a household’s annual food consumption.

### Catastrophic total costs due to TB

Catastrophic total costs due to TB is defined as total costs for TB treatment (the sum of direct and indirect costs for the entire TB episode) exceeding 20% of the household’s annual pre-TB income.

### Catastrophic health expenditure

CHE is defined OOP medical expenses for TB care exceeding a specified proportion of the household income or capacity to pay. There is no consensus on the threshold [9, 12]. In this study, we estimated CHE using two most commonly definitions: (a) annual OOP medical expenses over 40% of the household’s annual capacity to pay during TB treatment, and (b) annual OOP medical expenses over 10% of the household’s annual income during TB treatment [9, 12, 13].

### Basic medical insurance

Basic medical insurance is a social insurance system implemented by the government to compensate the economic losses caused by the disease risk of the insured. There are two types in Ningbo, one is the basic medical insurance for urban employees, which is mandatory and provided to people with a legitimate occupation. The other is the basic medical insurance for urban and rural residents, which is voluntary and covers mainly those under 18 years old, unemployed, and rural residents. Both are only valid at the place of enrollment.

### Rifampicin-susceptible (RS) pulmonary TB

RS pulmonary TB is confirmed by sputum culture of *Mycobacterium tuberculosis*, and clinical isolates are susceptible to rifampicin in an in vitro anti-TB drug sensitivity test.

### Rifampicin-monoresistant (RMR) pulmonary TB

RMR pulmonary TB is confirmed by sputum culture of *Mycobacterium tuberculosis*, and clinical isolates are resistant to rifampicin and susceptible to isoniazid in an in vitro anti-TB drug sensitivity test.

### Multidrug-resistant (MDR) pulmonary TB

MDR pulmonary TB is confirmed by sputum culture of *Mycobacterium tuberculosis*, and clinical isolates are resistant to both rifampicin and isoniazid in an in vitro anti-TB drug sensitivity test.

### Statistical analysis

Data were double entered and checked using EPI Data V3.1. Continuous variables were presented as means (standard deviation, SD) and medians (interquartile range, IQR), while categorical variables were described as absolute numbers and frequencies. Using univariate logistic regression analysis, the association between different factors and catastrophic total costs was examined by estimating odd ratios (ORs) with 95% confidence intervals (CIs). We used the least absolute shrinkage and selection operator (LASSO) regression for variable selection, and ten-fold cross-validation together with the 1SE rule were used to select the predictive variables from the primary data set. Multivariable analyses of selected variables related to catastrophic total costs were conducted using the logistic regression analysis. We addressed the interaction between hospitalization and severe adverse drug reaction by including the interaction term in a supplemental multivariable adjusted model. Adjusted odds ratios (aORs) and 95% confidence intervals were derived from the final model. The level of statistical significance was set at 5%. All analyses were performed using the statistical software R (http://www.R-project.org) and Empower (R) (www.empowerstats.com, X&Y Solutions, Inc., Boston, MA).

### Sensitivity analyses

We varied the threshold (20% ± 5%) to observe how this would affect the percentage of households incurring catastrophic total costs. We also compared the incidence of catastrophic total costs with different thresholds to the incidence of catastrophic health expenditures with two common definitions. Finally, a supplemental model was conducted including an interaction term between hospitalization and severe adverse drug reactions.

## Results

### Characteristics of participants and their households

A total of 683 patients were qualified, of whom 11 refused to participate. Thus, the analysis finally included 672 patients, of which 586 (87.2%) cases were RS, 30 (4.5%) were RMR, and 56 (8.3%) were MDR. Table 1 shows the basic characteristics and hospitalization of the participants. The average age of participants was 43 years, with a median age of 41 years. Approximately 90% of patients received at least primary school education. About one-third of patients were floating population and did not have health insurance where they lived or worked. Less than 5% of households lived below the poverty line in Ningbo. According to the World Bank’s poverty line standard of US $ 1.90 per day, 4.2% (28/672) of the households surveyed before suffering from TB were in poverty, and this proportion rose to 15.5% (104/672) after TB. The proportion of patients who were employed before illness was 62.8%, and after illness was 57.4%. Patients in the poorest households had the highest the hospitalization rates (Q1:54.8%, Q2:61.3%, Q3:52.2%, Q4:49.5%, Q5:69.7%, *P* = 0.011) and the highest incidence of severe adverse drug reactions (Q1:29.6%, Q2:19.6%, Q3:28.0%, Q4:33.7%, Q5:35.3%, *P* = 0.034).

**Table 1:** Sociodemographic and clinical characteristics of the study population, by hospitalization status and overall

Variables	Number of participants (%)	Hospitalisation (%)	Non-hospitalisation (%)	*P*-value*
Total participants	672 (100)	388 (57.7)	284 (42.3)	
Age (years)				0.030
< 60	514 (76.5)	285 (55.4)	229 (44.6)	
≥ 60	158 (23.5)	103 (65.2)	55 (34.8)	
Gender				0.376
Male	432 (64.3)	244 (56.5)	188 (43.5)	
Female	240 (35.7)	144 (60.0)	96 (40.0)	
Education				0.927
None	70 (10.4)	41 (58.6)	29 (41.4)	
Primary or Junior school	368 (54.8)	210 (57.1)	158 (42.9)	
Senior school or above	234 (34.8)	137 (58.5)	97 (41.5)	
Floating population				0.004
No	437 (65.0)	270 (61.8)	167 (38.2)	
Yes	235 (35.0)	118 (50.2)	117 (49.8)	
Minimum living security household				0.355
No	640 (95.2)	367 (57.3)	273 (42.7)	
Yes	32 (4.8)	21 (65.6)	11 (34.4)	
Residence				0.486
Rural	316 (47.0)	178 (56.3)	138 (43.7)	
Urban	356 (53.0)	210 (59.0)	146 (41.0)	
Employment status before illness				0.002
Employed	422 (62.8)	224 (53.1)	198 (46.9)	
Unemployed	250 (37.2)	164 (65.6)	86 (34.4)	
Employment status after illness				< 0.001
Employed	386 (57.4)	250 (64.8)	136 (35.2)	
Unemployed	286 (42.6)	138 (48.3)	148 (51.7)	
Local basic medical insurance				0.024
No	212 (31.5)	109 (51.4)	103 (48.6)	
Yes	460 (68.5)	279 (60.7)	181 (39.3)	
Household economic status				0.011
Q1	135 (20.1)	74 (54.8)	61 (45.2)	
Q2	163 (24.3)	100 (61.3)	63 (38.7)	
Q3	157 (23.4)	82 (52.2)	75 (47.8)	
Q4	95 (14.1)	47 (49.5)	48 (50.5)	
Q5	122 (18.2)	85 (69.7)	37 (30.3)	
Patient delay				0.263
No	312 (46.4)	173 (55.4)	139 (44.6)	
Yes	360 (53.6)	215 (59.7)	145 (40.3)	
Diagnosis delay				0.196
No	583 (86.8)	331 (56.8)	252 (43.2)	
Yes	89 (13.2)	57 (64.0)	32 (36.0)	
Type of case				0.002
New or never treated	601 (89.4)	335 (55.7)	266 (44.3)	
Relapse or previously treated	71 (10.6)	53 (74.6)	18 (25.4)	
Comorbidities				< 0.001
No	584 (86.9)	315 (53.9)	269 (46.1)	
Yes	88 (13.1)	73 (83.0)	15 (17.0)	
Seeking medical care outside the city				0.001
No	587 (87.4)	325 (55.4)	262 (44.6)	
Yes	85 (12.6)	63 (74.1)	22 (25.9)	
Severe adverse drug reactions				0.018
No	481 (71.6)	264 (54.9)	217 (45.1)	
Yes	191 (28.4)	124 (64.9)	67 (35.1)	
Drug sensitivity test				0.002
RS	586 (87.2)	324 (55.3)	262 (44.7)	
RMR	30 (4.5)	20 (66.7)	10 (33.3)	
MDR	56 (8.3)	44 (78.6)	12 (21.4)	

Note: Floating population refers to a group of migrants without Hukou registered in the place where they live or work

Minimum living security household is defined as a household’s anuual income per capita lower than US$ 1429. The designation of minimum living security household status needs to be certified by the local civil affairs bureau

Local basic medical insurance refers to the basic medical insurance registered in Ningbo city

Household economic status is ranked based on a household’s annual income per capita, with quartile 1 (Q1) being the richest and quartile 5 (Q5) being the poorest

Patient delay is determined as a time interval from the first onset of tuberculosis symptoms to the first visit to any health provider lasts for > 14 days

Diagnostic delay is defined as a time interval between a patient’s first visit to any health provider and the final diagnosis as TB lasts for > 14 days

Comorbidities refers to the simultaneous occurrence of malignancy, diabetes mellitus, renal disease, etc.

*RS* rifampicin-susceptible; *RMR* rifampicin-monoresistant; *MDR* multidrug-resistant

*the Chi-square tests were applied to assess differences between categories of variables

Total costs due to TB and its share of household’s pre-TB annual income among patients with RS, RMR and MDR were significantly different, but there was no statistical difference in the household’s pre-TB annual income. Among RS, RMR and MDR patients, the medical cost accounted for more than 60% of the total cost (Table 2).

**Table 2:** Total costs due to TB and household economic status, by drug susceptibility test

Variables	RS	RMR	MDR	*P*-value*
Total costs due to TB (US$)				
Mean (SD.)	2094.5 (2676.7)	7669.1 (9649.4)	10,802.1 (10,219.4)	
Median(p25,p75)	1377.8 (740.72503.7)	3622.2 (1159.312029.6) ^a^	7355.6 (3129.614255.6) ^a^	< 0.001
Medical costs share of total costs (%)				
Mean (SD.)	67.7 (24.4)	75.6 (24.1)	64.7 (31.1)	
Median(p25,p75)	70.8 (53.8,87.2)	79.9 (63.3,94.5)	75.7 (52.9,87.5)	0.155
Direct non-medical costs share of total costs (%)				
Mean (SD.)	25.4 (22.7)	20.4 (27.6)	27.9 (30.7)	
Median(p25,p75)	19.9 (8.5,34.6)	8.2 (3.1,24.4)	15.6 (8.6,35.1)	0.059
Indirect costs share of total costs (%)				
Mean (SD.)	7.7 (13.7)	6.9 (8.7)	11.0 (18.5)	
Median(p25,p75)	0.0 (0.0,11.4)	2.9 (0.0,13.9)	2.9 (0.0,14.7)	0.118
household’s pre-TB annual income (US$)				
Mean (SD.)	14,375.6 (10,762.1)	14,732.3 (6863.8)	14,510.6 (14,399.1)	
Median(p25,p75)	11,851.9 (7407.417777.8)	14,814.8 (8692.620729.6)	11,918.5 (8114.815633.3)	0.446
Total costs share of household’s pre-TB annual income (%)				
Mean (SD.)	21.6 (24.6)	29.5 (32.9)	46.3 (34.1)	
Median(p25,p75)	12.8 (5.7,26.8)	12.3 (5.2,50.1)	42.3 (14.0,74.4) ^a, b^	< 0.001

Note: US$: United States dollar

A currency exchange rate of Chinese RMB 675 Yuan to US$ 100 Yuan (at the end of 2017)

*RS* rifampicin-susceptible

*RMR* rifampicin-monoresistant

*MDR* multidrug-resistant

*Kruskal Wallis test

Pairwise comparisons were performed by post-hoc analyses with Bonferroni adjustment (3 comparisons: *P*-value< 0.017)

^a^significant compared to RS

^b^significant compared to RMR

Whether it was direct medical expenses or out-of-pocket medical expenses, inpatients were significantly higher than non-inpatients (Table 3).

**Table 3:** Direct medical expenses and medical costs, by hospitalization

Variables	Hospitalisation	Non-hospitalisation	*P*-value*
RS			
Direct medical expenses (US$)			
Mean (SD.)	3241.7 (3240.4)	1065.0 (1125.8)	
Median(p25,p75)	2437.0 (1670.43407.4)	859.3 (563.0,1255.6)	< 0.001
Medical costs (US$)			
Mean (SD.)	2094.7 (2623.8)	703.4 (994.9)	
Median(p25,p75)	1422.2 (825.92303.7)	518.5 (296.3959.3)	< 0.001
RMR			
Direct medical expenses (US$)			
Mean (SD.)	11,739.3 (8984.6)	2268.1 (2949.9)	
Median(p25,p75)	11,133.3 (4996.316174.1)	1540.7 (488.92440.7)	< 0.001
Medical costs (US$)			
Mean (SD.)	8460.0 (9204.2)	983.7 (971.3)	
Median(p25,p75)	4311.1 (2525.912111.1)	763.0 (92.61825.9)	< 0.001
MDR			
Direct medical expenses (US$)			
Mean (SD.)	12,559.6 (8252.9)	1363.0 (1033.6)	
Median(p25,p75)	11,348.1 (7103.716874.1)	1688.9 (511.11888.9)	< 0.001
Medical costs (US$)			
Mean (SD.)	9666.3 (8691.5)	855.6 (1033.3)	
Median(p25,p75)	7948.1 (3455.613151.9)	348.1 (0.0,1533.3)	< 0.001

Note: US$: United States dollar

A currency exchange rate of Chinese RMB 675 Yuan to US$ 100 Yuan (at the end of 2017)

*RS* rifampicin-susceptible

*RMR* rifampicin-monoresistant

*MDR* multidrug-resistant

*Mann-Whitney test

### Catastrophic total costs due to TB

Among the surveyed households, over 37% of households spent at least 20 of their household’s pre-TB annual income on pulmonary TB diagnosis and treatment. Table 4 presents how the incidence of catastrophic total costs changes across different thresholds. When the threshold varied from 25% of a household’s pre-TB annual income to 15%, the rate of catastrophic total costs increased rapidly from 30.2 to 46.0%. Regardless of the threshold, households affected by MDR always had the highest catastrophic cost rate, followed by RR and RS. The catastrophic cost rate calculated by using total costs over 15% or 20% of a household’s pre-TB annual income was close to the CHE rate measured by using OOP medical expenses over 40% of a household’s annual ability to pay during TB treatment.

**Table 4:** Comparison of the incidence of catastrophic total costs and CHE in PTB-affected households at different thresholds

Groups	the incidence of catastrophic total costs at the threshold			CHE1	CHE2
	15%	20%	25%		
RS (%)	43.3	33.6	26.3	39.6	56.0
RMR (%)	46.7	43.3	40.0	46.7	76.7
MDR (%)	73.2	69.6	66.1	71.4	91.1
Total (%)	46.0	37.1	30.2	42.6	59.8

Note: RS: rifampicin-susceptible

*RMR* rifampicin-monoresistant

*MDR* multidrug-resistant

CHE1: catastrophic health expenditure measured by annual out-of-pocket payments exceeding 40% of the household’s annual capacity to pay during TB treatment

CHE2: catastrophic health expenditure measured by annual out-of-pocket payments exceeding 10% of the household’s annual income during TB treatment

### The distributions of catastrophic total costs

In univariate logistic regression, many variables were statistically related to catastrophic total costs: age, education, minimum living security household, employment status before illness, employment status after illness, household economic status, patient delay, type of case, comorbidities, seeking medical care outside the city, hospitalization, severe adverse drug reactions, drug sensitivity test result (Table 5).

**Table 5:** Univariate logistic regression analysis of influencing factors of catastrophic total costs

Variables	n/N	%	OR	95% *CI*	*P*-value
Age (years)					
< 60	157/514	30.5	1		
≥ 60	92/158	58.2	3.17	2.19,4.58	< 0.001
Gender					
Male	161/432	37.3	1		
Female	88/240	36.7	0.97	0.70,1.35	0.877
Education					
None	38/70	54.3	1		
Primary or Junior school	155/368	42.1	0.61	0.37,1.02	0.062
Senior school or above	56/234	23.9	0.26	0.15,0.46	< 0.001
Floating population					
No	171/437	39.1	1		
Yes	78/235	33.2	0.77	0.55,1.08	0.129
Minimum living security household					
No	224/640	35	1		
Yes	25/32	78.1	6.63	2.82,15.58	< 0.001
Residence					
Rural	123/316	38.9	1		
Urban	126/356	35.4	0.86	0.63,1.18	0.344
Employment status before illness					
Employed	122/422	28.9	1		
Unemployed	127/250	50.8	2.54	1.83,3.51	< 0.001
Employment status after illness					
Employed	76/286	26.6	1		
Unemployed	173/386	44.8	2.24	1.61,3.12	< 0.001
Local basic medical insurance					
No	86/212	40.6	1		
Yes	163/460	35.4	0.80	0.58,1.12	0.201
Household economic status					
Q1	19/135	14.1	1		
Q2	35/163	21.5.	1.67	0.90,3.08	0.101
Q3	52/157	33.1	3.02	1.68,5.44	< 0.001
Q4	49/95	51.6	6.50	3.46,12.21	< 0.001
Q5	94/122	77.1	20.50	10.78,38.99	< 0.001
Patient delay					
No	101/312	32.4	1		
Yes	148/360	41.1	1.46	1.06,2.00	0.020
Diagnosis delay					
No	213/583	36.5	1		
Yes	36/89	40.4	1.18	0.75,1.86	0.477
Type of case					
New or never treated	206/601	34.3	1		
Relapse or previously treated	43/71	60.6	2.94	1.78,4.88	< 0.001
Comorbidities					
No	195/584	33.4	1		
Yes	54/88	61.4	3.17	2.00,5.03	< 0.001
Seeking medical care outside the city					
No	204/587	34.8	1		
Yes	45/85	52.9	2.11	1.34,3.34	0.001
Hospitalisation					
No	50/284	17.6	1		
Yes	199/388	51.3	4.93	3.42,7.09	< 0.001
Severe adverse drug reactions					
No	153/481	31.8	1		
Yes	96/191	50.3	2.17	1.54,3.05	< 0.001
Drug sensitivity test					
RS	197/586	33.6	1		
RMR	13/30	43.3	1.51	0.72,3.17	0.277
MDR	39/56	69.6	4.53	2.50,8.21	< 0.001

Note: Floating population refers to a group of migrants without Hukou registered in the place where they live or work

Minimum living security household, that is, a household’s anuual income per capita is less than US$ 1429, must be certified by the local civil affairs department

Local basic medical insurance refers to the basic medical insurance registered in Ningbo city

Patient delay is determined as a time interval from the first onset of tuberculosis symptoms to the first visit to any health provider lasts for > 14 days

Diagnostic delay is defined as a time interval between a patient’s first visit to any health provider and the final diagnosis as TB lasts for > 14 days

Household economic status is ranked based on a household’s annual income per capita, with quartile 1 (Q1) being the richest and quartile 5 (Q5) being the poorest

*RS* rifampicin-susceptible; *RMR* rifampicin-monoresistant; *MDR* multidrug-resistant

*OR* odds ratio; *95% CI* 95% confidence intervals

### Factors associated with catastrophic total costs

Based on nonzero coefficients in the LASSO regression model, thirteen possible variables affecting catastrophic total costs were included into the multivariable logistic regression model. In the adjusted model that controlled for confounding variables, the factors such as over 60 years old, minimum living security, unemployed before or after illness, poor economic status, seeking medical care outside the city, hospitalization, severe adverse drug reactions and MDR were positively associated with catastrophic costs. Local basic medical insurance was negatively related to catastrophic costs. Although preselected by LASSO to be related to catastrophic total costs, the factors “education”, “relapse or previously treated” and “comorbidities” were not found to be significant at the 5% level in the logistic regression model. We acknowledge the potential interaction between hospitalization and adverse drug reactions based on the theoretical framework, therefore we included the term into our final model to improve the model fit. The results indicated that, except for adverse drug reactions, the others were similar to the original findings (Table 6).

**Table 6:** Multivariable logistic regression analysis of factors associated with catastrophic total costs

Variable	Multivariable			Multivariable*		
	aOR	95% *CI*	*P*-value	aOR	95% *CI*	*P*-value
Age (years)						
< 60	1			1		
≥ 60	3.41	1.85,6.32	< 0.001	4.32	2.26,8.27	< 0.001
Education						
None	1			1		
Primary or Junior school	1.31	0.62,2.80	0.480	1.31	0.62,2.80	0.480
Senior school or above	1.09	0.46,2.62	0.840	1.09	0.46,2.62	0.840
Minimum living security household						
No	1			1		
Yes	5.69	1.95,16.60	0.001	5.69	1.95,16.60	0.001
Employment status before illness						
Employed	1			1		
Unemployed	1.67	1.04,2.69	0.036	1.67	1.04,2.69	0.036
Employment status after illness						
Employed	1			1		
Unemployed	2.47	1.49,4.08	< 0.001	2.47	1.49,4.08	< 0.001
Local basic medical insurance						
No	1			1		
Yes	0.52	0.32,0.85	0.009	0.52	0.32,0.85	0.009
Household economic status						
Q1	1			1		
Q2	1.54	0.75,3.17	0.242	1.54	0.75,3.17	0.242
Q3	3.00	1.48,6.10	0.002	3.01	1.48,6.10	0.002
Q4	7.28	3.29,16.10	< 0.001	7.26	3.28,16.06	< 0.001
Q5	15.84	7.27,34.55	< 0.001	15.91	7.28,34.76	< 0.001
Type of case						
New or never treated	1			1		
Relapse or previously treated	1.35	0.70,2.61	0.376	1.35	0.70,2.61	0.378
Comorbidities						
No	1			1		
Yes	1.71	0.94,3.13	0.080	1.71	0.94,3.12	0.082
Seeking medical care outside the city						
No	1			1		
Yes	2.29	1.21,4.34	0.011	2.29	1.21,4.34	0.011
Hospitalisation						
No	1			1		
Yes	5.42	3.35,8.76	< 0.001	5.54	3.15,9.75	< 0.001
Severe adverse drug reactions						
No	1			1		
Yes	1.95	1.24,3.08	0.004	2.05	0.90,4.66	0.087
Drug sensitivity test						
RS	1			1		
RMR	1.15	0.45,2.97	0.768	1.16	0.45,2.99	0.761
MDR	2.78	1.28,6.02	0.010	2.77	1.27,6.01	0.010

Note: Minimum living security household is defined as a household’s anuual income per capita lower than US$ 1429. The designation of minimum living security household status needs to be certified by the local civil affairs bureau

Local basic medical insurance refers to the basic medical insurance registered in Ningbo city

Household economic status is ranked based on a household’s annual income per capita, with quartile 1 (Q1) being the richest and quartile 5 (Q5) being the poorest

Comorbidities refers to the simultaneous occurrence of malignancy, diabetes mellitus, renal disease, etc.

*RS* rifampicin-susceptible; *RMR* rifampicin-monoresistant; *MDR* multidrug-resistant

*aOR* Adjusted odds ratio; *95% CI* 95% confidence intervals

*The multivariable logistic model accounted the interaction between hospitalization and severe adverse drug reactions

## Discussion

Putting an end to the catastrophic total costs due to TB is one of the three goals of the End TB strategy by WHO [2, 3, 14]. In this study examining the catastrophic total costs due to TB among patients treated in the NTCP network under a designated hospital service model, we found that more than one-third of patients and their households who were diagnosed and treated in designated hospitals face financial disasters due to TB, especially those with MDR. The incidence of catastrophic costs in this survey is lower than those of Ghana [15] and Vietnam [16], and is similar to the results of studies in Indonesia [17], Peru [18], and southern India [19].

We identified several independent factors associated with catastrophic total costs in this study, these factors could have influenced healthcare costs through different mechanisms. For example, patients over 60 years old, unemployment before illness, minimum living security status and lower income households may correlate with household’s annual pre-TB income. On the other hand, lack of basic medical insurance coverage, seeking medical treatment outside the city, hospitalization and MDR affect direct costs, and unemployment after illness are associated with income losses. These findings highlighted that in addition to reducing the direct costs of TB care for patients, especially those with MDR and severe adverse drug reactions [15], other financial protection measures such as compensation for income losses were needed to prevent low-income households from incurring catastrophic costs following TB diagnosis [2, 14, 16].

Consistent with studies in other countries [17–19], there was an obviously negative correlation between the household’s economic status and the occurrence of catastrophic total costs, that is, financially vulnerable households were more likely to suffer catastrophic costs. This prompted us to think about the drawbacks of the balanced subsidy policy. While the governments distribute TB subsidies the restricted budget every year, “one-size-fit-all” subsidies ensure the fairness of the program, but it ignored the disparate financial vulnerability among patients and households with TB [9, 14, 20, 21]. Meanwhile, a more precise, tailored financial support might play a better role in poverty alleviation by minimizing the rate of catastrophic costs. Public subsidies programs often face budgetary constraints, a differentiated subsidy policy based on the household’s economic status has more potential prioritizing protection to the poor against catastrophic costs.

According to the recommendations of the WHO and China’s National TB Control Program, outpatient treatment is usually advocated for non-severe and/or non-MDR cases [22]. Nevertheless, the survey found that more than half of the patients were hospitalized during diagnosis and treatment, which is in line with other studies conducted in China [9, 23]. Previous studies have shown that hospitalization will lead to a substantial increase in the cost of treatment, and hospitalization cases face higher economic risks than outpatient cases [6, 22]. In this study, hospitalization was a key factor influencing catastrophic costs, and its impact was second only to the household economic status. In recent years, as the responsibility for TB care has shifted from the Center for Disease Control and Prevention (CDC) to general hospitals, the proportion of patients hospitalized has increased significantly [9, 24, 25]. A literature search indicated that unnecessary hospitalization for TB is common in China, which is considered to be closely related to the profit-seeking behavior of the hospital [5, 6, 26–28]. This behavior also reasonably explains that the direct medical costs borne by patients receiving treatment in designated hospitals in Ningbo were much higher than those reported in other countries [16, 17, 19], whether they were absolute values or their share of total costs.

Our findings have at least three policy implications. First, the existing “zero OOP” free TB care policy developed under the CDC service model implemented since the 1990s was insufficient in addressing the problem of catastrophic costs faced by patients. As our findings indicated, approximately 40% of individuals and their households incurred catastrophic spending due to TB. Secondly, we documented inefficiency in TB care delivery system. Despite adoption of economic protection interventions such as universal health coverage and cash transfers, patients and national TB program are challenged by excessive costs caused by unnecessary hospitalization, out-of-network medical care and inappropriate prescribing patterns. Healthcare facility managers and government administrators should conduct in-depth investigation into the causes of excessive direct medical costs, as well as audit to identify fraud and adverse financial incentives to physicians. Additionally, fee-for-service payment model could have incentivized unnecessary care and overprescribing of certain medications. Value-based payment model with outcome-based contracting with TB treatment services might play a role in reducing overall costs without impacting care quality. Finally, there is reason to believe that the current one-size-fit all subsidy policy may be inadequate for the most financially vulnerable population. Policymakers should consider tailored financial subsidies addition to in-kind support to improve equity of TB programs. On the other hand, means test and asset assessment should be considered in determining program eligibility to improve the efficiency of subsidies programs.

Our study has some limitations. First, the study did not include some patients who did not receive treatment or discontinued treatment prematurely. If this situation is caused by financial barriers, it has the possibility of underestimating the actual level of catastrophic costs. However, the research still illustrates that patients and their households face huge economic risks. Second, we employed self-reported actual medical costs (cross-referenced by the medical receipts collected by community doctors during each visit) to estimate the cost estimates in this study rather than the method recommended by WHO, which suggests asking these questions while on treatment. We adopted this approach based on the operational reasons since it was logistically challenging to track and survey internal migrant patients on treatment. Our approach may have introduced recall bias in economic outcome measures. However, in the pilot survey, we found that our approach and WHO-recommended method yielded similar cost estimates in a subgroup of study participants. Nevertheless, we acknowledge the recall bias on self-reported direct non-medical expenses, household income and food expenditure due to longer recall time as one of the limitations.

## Conclusion

In Ningbo, households with pulmonary TB patient treated in designated hospitals, especially those with MDR, were facing catastrophic economic risks. There were many independent factors that affected the occurrence of catastrophic costs. Although direct medical costs accounted for most of the catastrophic costs, protecting patients from financial disasters requires not only expanding universal health coverage, but also more personalized economic protection measures such as cash transfers to compensate low-income households for direct non-medical expenses and income loss. In addition, attention should be paid to payment model reforms aimed at reducing unnecessary hospitalization.

## Data Availability

Please contact the corresponding author for data requests.

## Abbreviations

TB: Tuberculosis
CHE: Catastrophic Health Expenditure
WHO: World Health Organization
NTCP: National Tuberculosis Control Program
OOP: Out-of-pocket
RS: Rifampicin-Susceptible
RMR: Rifampicin-Monoresistant
MDR: Multidrug-Resistant
LASSO: Least Absolute Shrinkage and Selection Operator
